# Hospital Housekeeping

**Published:** 1898-12-24

**Authors:** 


### HOSPITAL HOUSEKEEPING.

By a late Matron.

### IV.—CHIEFLY ABOUT BREAD AND BUTTER.

In touring about the Continent, visiting—as every good nurse does—any and all of the hospitals and institutions to which I could gain admission, I have invariably been impressed with the talent for catering exhibited in foreign hospitals. Many times in France I have been tempted to envy the patients their sojourn in hospital, including, as this did, a steady consumption of delicious, crisply-baked rolls which, with a generous pat of the sweet, creamy butter produced in Normandy and Brittany, comprise in themselves a delightful dietary. Contrast such bread and such butter with the stodgy, ill-baked "household" loaf served in the average English hospital, with a "scrape" of the inferior butter so often served in the ward, and it will be at once evident that in these two particulars, where we so conspicuously fail, they manage much better in France. I am at a loss to understand why all our hospitals do not make their own bread. On so large a scale it would not only prove economical, but absolute purity and freedom from alum and all the other deleterious substances wherewith the baker "drugs" his dough would be ensured. The bread would be well baked, for nobody then would profit by the wet half-cookedness which adds so materially to the weight, and to the baker's profit. Nobody with an elementary knowledge of dietetics will for one moment deny that the bread we eat, even in health, is of the utmost importance to the well-being of our digestions, and internal processes generally. To the sick, especially in the medical wards, the purity of bread and the absence of sourness and fermentative processes in the "staff of life," is of such paramount importance that one cannot for a moment understand why contract baker's bread is ever served in our hospitals. But a still greater element of wonder rests in the circumstance that even if "baker's bread" be allowed, why is there not a more efficient supervision as to the composition and general standard of "ward bread"?

As a practical breadmaker I approach this subject more confidently, and I must confess to having administered with grave misgivings the stodgy, sour bread compound which may be seen any day in several of our leading hospitals, to cases of convalescent typhoid gastritis, and enteritis.

For such cases the average "bread of commerce" is by no means suitable; neither is it scientific to serve such bread in a medical ward side by side with the most carefully prepared meat juices, peptonised foods, and delicate broths. Such bread is capable of acting as a very serious antidote to the scheme of delicate dietetics which is beginning to mark English treatment. It would certainly be a wise provision in every hospital for some one person to be held responsible for a general dietetic excellence, and the first reform a properly trained inspector of hospital dietaries would introduce would be a "bread reform."

The bread of average English domestic life is notoriously bad. In large towns special breads are manufactured and sold—at "special" prices. But the bulk of Britishers eat inferior bread. So long as they are healthy and satisfied with inferior bread I have no criticism to offer; but in the interests of hospital housekeeping I must maintain that the bread supplied to the sick in our wards should be home-made and above suspicion.

The argument used against home-made bread is that bread-making is so troublesome. As a matter of fact the manufacture of bread is a very simple matter, and, even if it were not so, the argument of "trouble" has no place in the dieting and care of the sick. If "trouble" is going to add a new healing factor to those already at command, we must welcome the labour with open arms.

In our prisons the bread is invariably baked on the spot, and is usually most excellent in flavour and quality. A special "baking" is prepared for prison infirmary use, and a better bread than that served to the sick prisoners no person could wish to have. Probably the reason why prison bread is home-made is owing to the circumstance that prisoners living on restricted diet need so staple a food as bread to be of the most excellent quality. So, too, do our sick.

Added to the argument that sick people need the very best quality of food, is the circumstance that persons lying inactive in bed cannot digest a fair quantum of bread unless the starchy elements thereof be specially well cooked. In short, it seems like "carrying coals to Newcastle" to insist further on the self-evident necessity of providing sick people with pure, wholesome crisp bread, instead of the damp, heavy, half-cooked compound which so often does duty in our hospital wards.

For those who cannot visit the Continental hospitals for an object lesson in bread and butter, the French Hospital in London will afford an admirable example of these, and will furnish an eloquent type of an excellent standard of hospital cuisine achieved with economy.

I remember once in France, when I was praising the admirable dietetic achievements in a hospital, carried out as these were at half the cost which we in England should consider necessary to attain such a result, the Superintendent Sister, who had been for many years in a London hospital, said "I would undertake to almost feed my hospital from the amount wasted in a similar London institution," and there must be a great deal of truth in her argument, since the prices of most articles of food in England are far below those of France, despite which practical fact she was able to feed her patients admirably at a cost per capita which we should regard as starvation rate.

Now let us go into this waste question, beginning with the bread and butter department—items which add up to serious proportions in hospital accounts.

I have interested myself on several occasions in inspecting the amount of bread wasted daily in some of our London hospitals, and I must confess that the resulting total has appeared alarming to an economist. The worst of it is that the amount of waste is rarely gauged. Slices of bread are thrown in with ashes and ward refuse. I have seen them in the receptacles provided for waste surgical dressings, poultices, &c.; and wardmaids have been seen to throw "left over" food on to the kitchen fire.

Now, as to the waste of butter, we know that butter is not sent up to our wards in too generous quantities, and there does not appear much margin for waste in this expensive item of food. No; but the waste consists in the indiscriminate serving of so many slices of spread bread and butter to each patient. Some sick people take just one mouthful—the remainder is thrown away. Discretion in serving out the food would prevent this daily waste—a waste which in individual wards every day does not appear very flagrant. But calculate the daily waste in all the wards in the hospital, multiply the result by 365, and the annual sum is appalling. In the days of my matronship I found the remedy in a simple expedient. I established in every ward a clean wicker basket into which every stray piece of bread or bread and butter was put. I also insisted that the waste food from all plates should be put into a tin receptacle kept entirely for such refuse. A periodic inspection of these revealed the "wasteful wards," and the sisters and ward nurses thereof were given to understand that they were held personally responsible for every slice of bread and piece of good wholesome food which found its way unnecessarily to the waste tins and baskets.

